# Defects in MAP1S-mediated autophagy turnover of fibronectin cause renal fibrosis

**DOI:** 10.18632/aging.100957

**Published:** 2016-05-25

**Authors:** Guibin Xu, Fei Yue, Hai Huang, Yongzhong He, Xun Li, Haibo Zhao, Zhengming Su, Xianhan Jiang, Wenjiao Li, Jing Zou, Qi Chen, Leyuan Liu

**Affiliations:** ^1^ Department of Urology, The Fifth Affiliated Hospital of Guangzhou Medical University, Guangzhou, Guangdong, 510700, China; ^2^ Center for the Innovation and Translation of Minimally Invasive Techniques, Guangzhou Medical University, Guangzhou, Guangdong, 510700, China; ^3^ Institute of Biosciences and Technology, Texas A&M Health Science Center, Houston, TX 77030, USA; ^4^ Department of Urology, The Sun Yat-sen Memorial Hospital, Sun Yat-sen University, Guangzhou, Guangdong, 510120, China; ^5^ Guangdong Provincial Key Laboratory of Malignant Tumor Epigenetics and Gene Regulation, Sun Yat-Sen Memorial Hospital, Sun Yat-Sen University, Guangzhou, Guangdong, 510120, China; ^6^ Department of Molecular and Cellular Medicine, College of Medicine, Texas A&M Health Science Center, TX 77843, USA

**Keywords:** autophagy, caspase 1, collagen, fibronectin, inflammasome, MAP1S, pyroptosis, renal atrophy, renal fibrosis

## Abstract

Excessive deposition of extracellular matrix proteins in renal tissues causes renal fibrosis and renal function failure. Mammalian cells primarily use the autophagy-lysosome system to degrade misfolded/aggregated proteins and dysfunctional organelles. MAP1S is an autophagy activator and promotes the biogenesis and degradation of autophagosomes. Previously, we reported that MAP1S suppresses hepatocellular carcinogenesis in a mouse model and predicts a better prognosis in patients suffering from clear cell renal cell carcinomas. Furthermore, we have characterized that MAP1S enhances the turnover of fibronectin, and mice overexpressing LC3 but with MAP1S deleted accumulate fibronectin and develop liver fibrosis because of the synergistic impact of LC3-induced over-synthesis of fibronectin and MAP1S depletion-caused impairment of fibronectin degradation. Here we show that a suppression of MAP1S in renal cells caused an impairment of autophagy clearance of fibronectin and an activation of pyroptosis. Depletion of MAP1S in mice leads to an accumulation of fibrosis-related proteins and the development of renal fibrosis in aged mice. The levels of MAP1S were dramatically reduced and levels of fibronectin were greatly elevated in renal fibrotic tissues from patients diagnosed as renal atrophy and renal failure. Therefore, MAP1S deficiency may cause the accumulation of fibronectin and the development of renal fibrosis.

## INTRODUCTION

Mammalian cells primarily use the autophagy-lysosome pathway to degrade dysfunctional organelles, misfolded/aggregated proteins and other macro-molecules [1]. After being translated and exported to the surface of plasma membrane through exocytosis [2], fibronectin initiates the assembly of fibronectin extracellular matrix and other extracellular matrix proteins such as collagen [3]. Following endocytosis, fibronectin is packaged into early endosome, matured to late endosome and directly degraded in lysosomes [4, 5]. Autophagy defects lead to impairment of fibro-nectin degradation and excessive deposition of fibronectin as extracellular matrix, which leads to renal fibrosis [6]. On the other hand, autophagy defects lead to enhancement of oxidative stresses [1, 7]. Oxidative stress in turn activate NLRP3 inflammasome that result in a direct activation of caspase-1 and generation of P10 form of caspase 1 [8]. The activation of caspase-1 subsequently induces secretion of potent pro-inflammatory cytokines interleukin-1β (IL-1β) and IL-18, mitochondrial dysfunction, production of excess reactive oxygen species, and eventually an inflammatory form of cell death referred as pyroptosis [9–14]. Pyroptotic cells release pro-inflammatory signals to promote the mortality and impair the survival of host structural, hematopoietic and immune-competent cells [8, 11, 14, 15]. Inflammation-induced renal tissue remodeling promotes the production of fibronectin to boost renal fibrosis [16, 17].

MAP1S, previously named as C19ORF5, is a member of the microtubule-associated protein family 1. Similar to its homologues MAP1A and MAP1B, MAP1S interacts with both LC3-I and LC3-II isoforms [18–22]. We identified MAP1S as a positive regulator of autophagy and its depletion led to autophagy defects under nutritive stress and an accumulation of dysfunctional mitochondria [22]. The general MAP1S knockout mice exhibit impaired degradation of fibronectin, increased intensities of sinusoidal dilatation and increased levels of oxidative stress in liver, and reduced lifespans. Overexpression of fibronectin generates a stress so that the knockout mice develop liver fibrosis and live further shortened lifespans [5]. In addition to other types of cancers such as ovarian cancer [23], hepatocellular carcinomas [24], human prostatic adenocarcinomas [25] and pancreatic ductal adenocarcinomas [26], we found that MAP1S-mediated autophagy facilitates turnover of lipid droplets to suppress the development of clear cell renal cell carcinomas (ccRCC) and similarly promotes the survival of cancer patients [27]. Because of the involvement of MAP1S in both liver fibrosis and ccRCC, we were triggered to investigate the roles of MAP1S in renal fibrosis. In our current study, we found that MAP1S-mediated autophagy promoted the turnover of fibronectin and suppressed pyroptosis in normal renal cells. MAP1S deficiency led to accumulation of fibronectin and development of renal fibrosis in both mice and human beings.

## RESULTS

### Levels of fibronectin are elevated and levels of MAP1S are decreased in renal tissues from patients suffering from renal fibrosis

**Figure 1:**
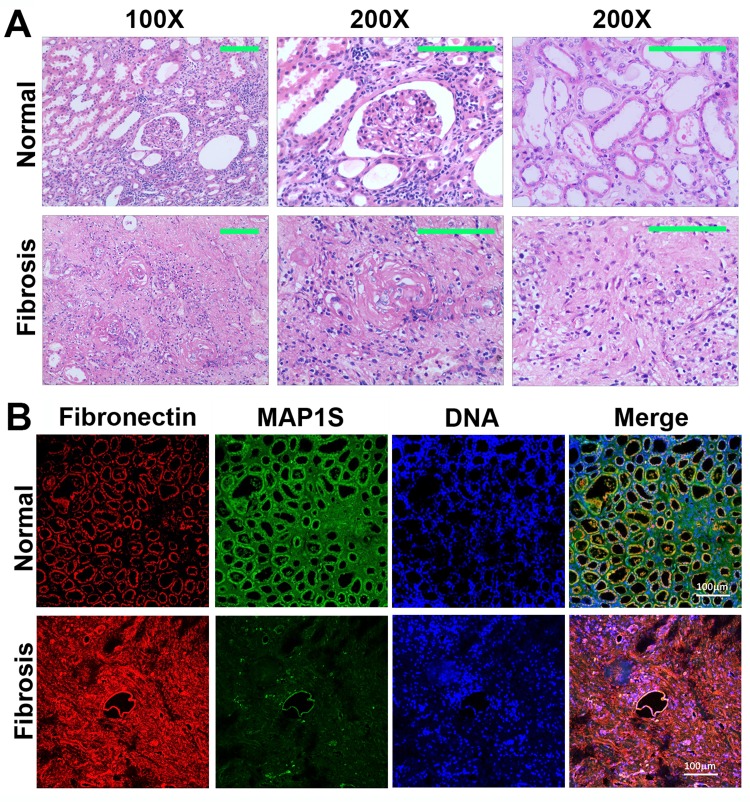
Levels of MAP1S are decreased and levels of fibronectin are elevated in renal tissues from patients suffering from renal fibrosis (**A**) Representative images showing the H&E staining of renal tissues collected from patients suffering from renal fibrosis and healthy control. The area containing a glomerulus or distal and proximal convoluted tubules is amplified (200X) to show the detail structures. (**B**) Representative images showing the immuno-florescent staining of MAP1S (green), fibronectin (red) and nuclear DNA (blue) in the normal and renal fibrotic tissues. Scale Bar: 100 μm.

Six patients diagnosed as renal atrophy and renal failure and six normal controls were subjected to analyses of renal fibrosis by H&E staining. We found that the areas containing a glomerulus or distal and proximal convoluted tubules exhibited disorganized renal structures and were filled with fibrotic tissues (Fig. 1A).

We further conducted immuno-fluorescent staining and revealed that levels of fibronectin were dramatically elevated while the levels of MAP1S were dramatically reduced (Fig. 1B). Therefore, high levels of fibronectin are associated with low levels of MAP1S in patients suffering from renal fibrosis.

### MAP1S reduces levels of fibronectin through autophagy

**Figure 2:**
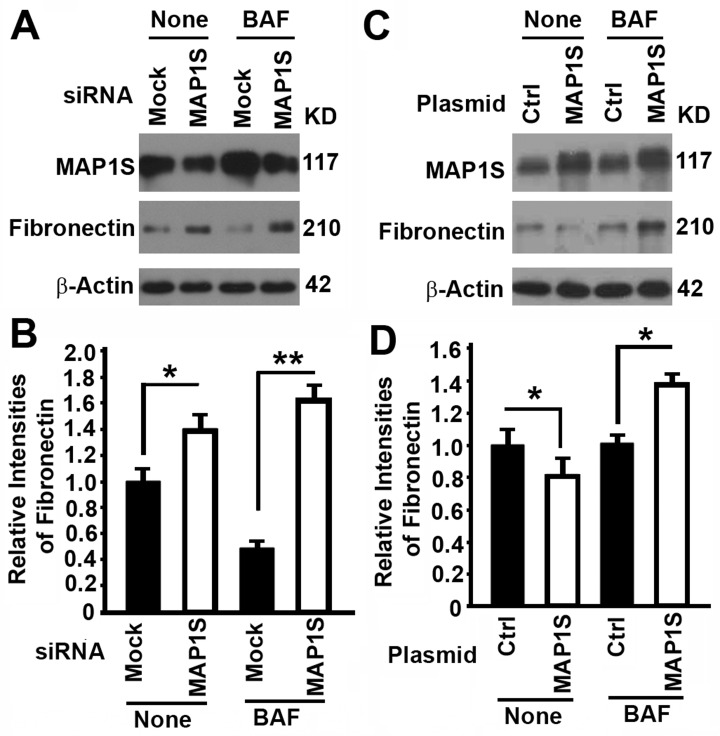
The impact of MAP1S on the levels of fibronectin in HK2 cells (**A‐D**) Representative immunoblot images (**A,C**) and plots (**B,D**) showing the impact of MAP1S suppression (**A,B**) or overexpression (**C,D**) on the levels of fibronectin in the absence (None) or presence of bafilomycin A1 (BAF). Bars in panels (**B,D**) represent mean ± standard deviation of fibronectin levels between different groups. The significance is estimated by Student's T Test with two‐tailed distribution and unequal variances. *, p < 0.05; ** and p < 0.01.

We have shown that MAP1S facilitates the turnover of fibronectin through lysosomes in liver tissues and suppresses liver fibrosis in mouse models [5]. We altered the expression of MAP1S in HK2 cells, a proximal tubular cell (PTC) line derived from normal kidney and immortalized by transduction with human papillomavirus 16 (HPV-16), to test its impact on the levels of fibronectin. Suppression of MAP1S with MAP1S-specific siRNA caused the accumulation of fibronectin in the absence of lysosomal inhibitor bafilomycin A1 (BAF) (Fig. 2A, B), suggesting an inhibition of lysosomal degradation. Overexpression of MAP1S with a plasmid carrying MAP1S caused a reduction in levels of fibronectin in the absence of BAF (Fig. 2C,D), suggesting either a reduction of fibronectin synthesis or an activation of lysosomal degradation. Accumulation of high levels of fibronectin in the presence of BAF indicated that it was more likely an activation of lysosomal degradation (Fig. 2C,D). Interestingly, the levels of fibronectin in cells with either MAP1S suppressed or MAP1S overexpressed were higher than those in the controls (Fig. 2), suggesting an BAF plays an additional unknown role on fibronectin in addition to its inhibition of lysosomal activity. Thus, MAP1S promotes the turnover of fibronectin through lysosomes.

### Depletion of MAP1S causes accumulation of fibronectin and renal fibrosis in aged mice

**Figure 3:**
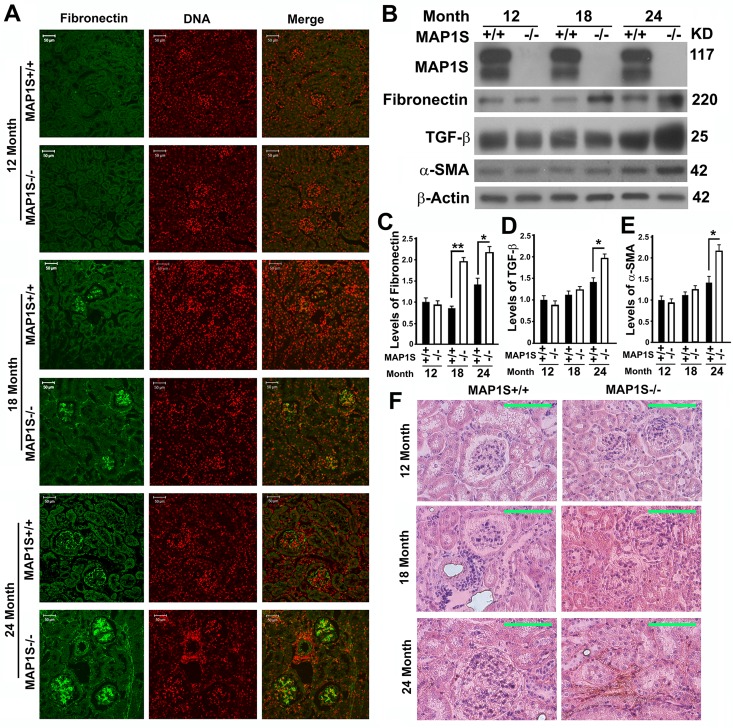
Depletion of MAP1S causes accumulation of fibronectin and renal fibrosis in aged mice (**A**) Immunostaining analyses of fibronectin (green) in sections from renal tissues renal tissues collected from wild-type and MAP1S^−/−^ mice at different ages using anti-fibronectin antibody. Nuclear DNA is counter-stained as red. Bar: 50 μm. (**B-E**) Representative immunoblot images (**B**) and plots (**C-E**) showing the impacts of MAP1S on the levels of fibronectin (**C**), TGF-β (**D**) and α-SMA (**E**) in renal tissues described in panel (**A**). The initial intensity of each protein in the 12-month-old wild-type was set to be 1. Data shown in plots above were the averages and standard deviations of three repeats. Plots were the means ± S.D. of three repeats and the significance of the differences was compared as described above. (**F**) Comparative Sirius Red staining of renal tissues described in panel (**A**). Bar = 100 μm.

To further compare the impact of MAP1S on the levels of fibronectin, we collected the renal tissue samples from 12, 18 and 24 month-old wild-type and MAP1S knockout mice. Based on immunostaining analyses of fibronectin, we observed no obvious difference between wild-type and MAP1S^−/−^ mice at the age of 12 months but dramatic differences between wild-type and MAP1S^−/−^ mice at the age of 16 and 24 months (Fig. 3A). Such differences in the levels of fibronectin were further confirmed by immunoblot analyses (Fig. 3B,C). Further examination of fibronectin staining in detail revealed that some signals displayed in fibrillary structures appeared in the 24 month-old MAP1S^−/−^ mice (Fig. 3A). The suggested renal fibrosis in aged MAP1S^−/−^ mice by the fibronectin staining was further confirmed by immunoblot analyses of the levels of fibrosis-related proteins TGF-β and α-SMA (Fig. 3B,D,E) and Sirius Red staining (Fig. 3F). Therefore, MAP1S depletion causes renal fibrosis in aged mice.

### Autophagy defects triggered by MAP1S deficiency cause accumulation of fibronectin in mouse renal tissues

**Figure 4:**
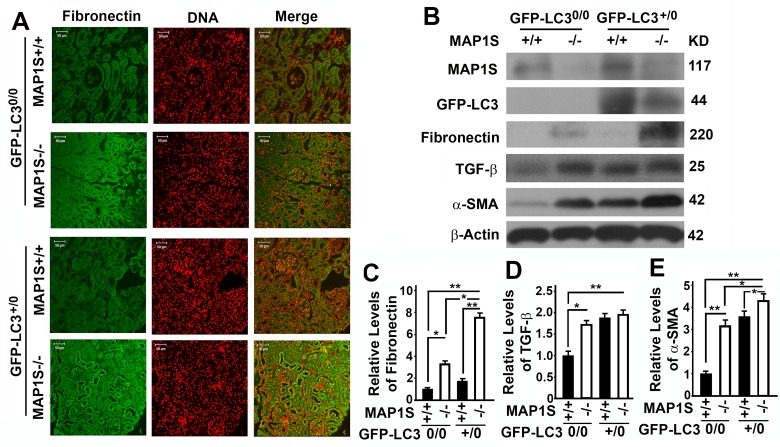
Autophagy defects triggered by MAP1S deficiency cause accumulation of fibronectin in mouse renal tissues (**A**) Immunostaining analyses of fibronectin (green) in renal tissues collected from 6-month-old wild-type (MAP1S^+/+^:GFP-LC3^0/0^), knockout (MAP1S^−/−^:GFP-LC3^0/0^), GFP-LC3 transgenic (MAP1S^+/+^:GFP-LC3^+/0^) and MAP1S^−/−^:GFP-LC3^+/0^ mice using anti-fibronectin antibody. Nuclear DNA is counter-stained as red. Bar: 50 μm. (**B-E**) Representative immunoblot images (**B**) and plots (**C-E**) showing the impacts of MAP1S on the levels of fibronectin (**C**), TGF-β (**D**) and α-SMA (**E**) in renal tissues described in panel (**A**). The initial intensity of each protein in the wild-type was set to be 1. Data shown in plots above were the averages and standard deviations of three repeats. Plots were the means ± S.D. of three repeats and the significance of the differences was compared as described above.

We reported that overexpression of GFP-LC3 leads to over-synthesis of fibronectin in hepatocytes [5]. In contrast, we observed no much difference in fibronectin between wild-type mice expressing and not expressing GFP-LC3 (Fig. 4A). However, levels of fibronectin were elevated in the renal tissues from MAP1S^−/−^ mice, and such elevation of fibronectin levels was enhanced in MAP1S^−/−^ mice expressing GFP-LC3 (Fig. 4A-C). Although no renal fibrosis was observed in such young mice, levels of fibrosis-related proteins TGF-β and α-SMA were increased due to MAP1S depletion (Fig. 4B,D,E).

### MAP1S suppresses pyroptosis in HK2 cells

**Figure 5:**
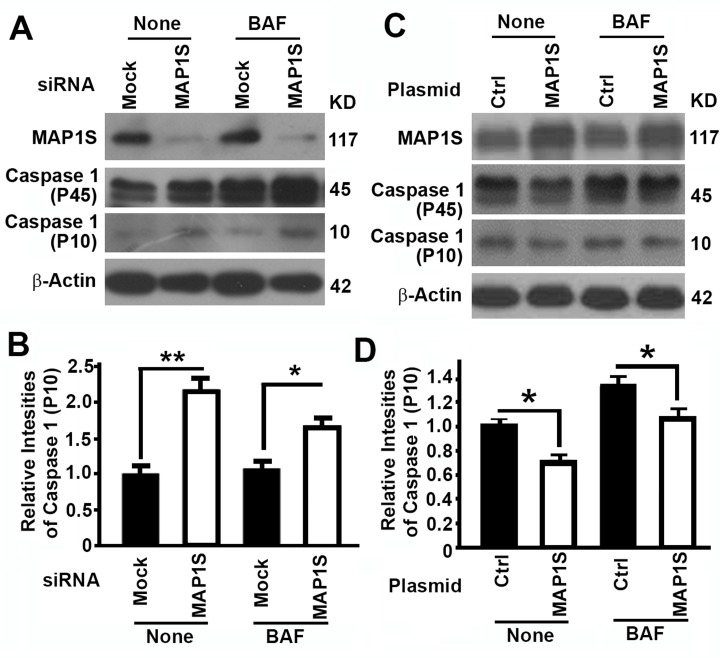
The impact of MAP1S on the levels of pyroptosis in HK2 cells (**A-D**) Representative immunoblot images (**A,C**) and plots (**B,D**) showing the impact of MAP1S suppression (**A,B**) or overexpression (**C,D**) on the levels of caspase 1 (P10) in the absence (None) or presence of bafilomycin A1 (BAF). Bars in panels (**B,D**) represent mean ± standard deviation of levels of caspase 1 P10 between different groups. Significance is estimated as described above.

To further understand the mechanism by which MAP1S affects renal fibrosis, we tested the impact of MAP1S on the levels of pyroptosis. Suppression of MAP1S with siRNA caused an increase and overexpression of MAP1S caused a suppression of caspase 1 P10 in HK2 cells although the impacts of MAP1S suppression and overexpression on caspase 1 P45 were not dramatic (Fig. 5). Thus, MAP1S suppresses pyroptosis.

## DISCUSSION

**Figure 6:**
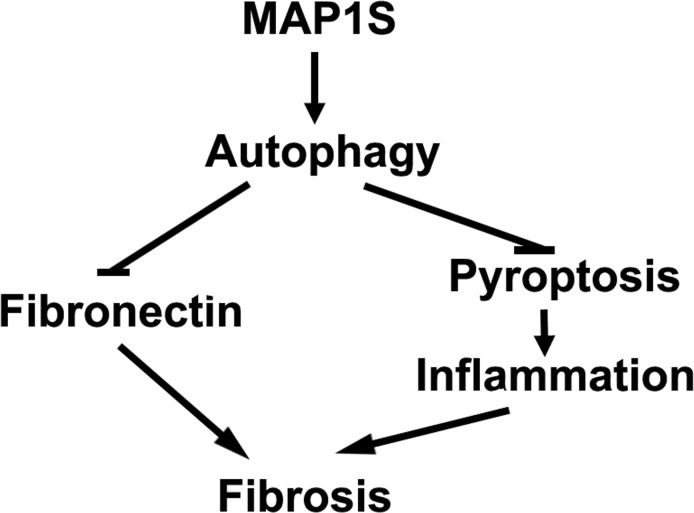
MAP1S-activated autophagy suppresses tissue fibrosis A diagram showing MAP1S activates autophagy to directly suppress fibrosis by promoting the turnover of fibronectin and indirectly impact fibrosis by suppressing pyroptosis and inflammation.

Autophagy defect has been implicated in disorders characterized by fibrosis in various tissues including renal fibrosis [28]. It can directly lead to excessive deposition of extracellular matrix such as fibronectin to initiate renal fibrosis or indirectly activate renal fibrogenesis by enhancing oxidative stress (Figure 6) [28]. Oxidative stress triggered by autophagy defects induces cell death, including apoptosis, necrosis and pyroptosis [29, 30]. Pyroptosis is specifically characterized by the activation of caspase-1 and release of pro-inflammatory cytokines to stimulate sterile inflammation and cause death of other cells in the environment [31]. Consequently, renal regeneration is activated to compensate the cellular loss. MAP1S is an activator of autophagy flux [22]. It not only activates general autophagy to suppress oxidative stress but also specifically promotes the lysosomal turnover of fibronectin [5, 22]. We have already reported that MAP1S knockout mice develop liver fibrosis and sinusoidal dilation in liver tissues when mice are under the stress of excessive production of fibronectin induced by LC3 [5]. Similarly, we observed similar consequence of MAP1S depletion in renal tissues. We reported that MAP1S enhances the clearance of lipid droplets through autophagy, which leads to suppression of ccRCC [27]. Here we also show that MAP1S enhances the lysosomal turnover of fibronectin and suppresses pyroptosis in renal cells and tissues. MAP1S depletion eventually causes renal fibrosis in aged mice. Therefore, MAP1S promotes autophagy and suppresses renal fibrosis.

In response to renal tissues injury, a temporary fibronectin scaffold containing plasma fibronectin originated from hepatocytes and cellular fibronectin produced locally will be formed to initiate renal tissue regeneration [32]. GFP-LC3-induced overexpression of fibronectin leads to accumulation of high levels of fibronectin in liver tissues from both wild-type and MAP1S^−/−^ mice [5]. However, the levels of fibronectin in renal tissues from wild-type mice expressing GFP-LC3 were actually lower than those from MAP1S^−/−^ mice not expressing GFP-LC3 (Fig. 4B,C), suggesting that MAP1S-mediated autophagy flux facilitated the efficient degradation of fibronectin in renal tissues so that no fibronectin accumulated. Defective autophagy flux in MAP1S^−/−^ mice lead to accumulation of more fibronectin in MAP1S^−/−^ mice either expressing GFP-LC3 or not. Therefore, MAP1S-mediated autophagy helps renal tissues to maintain low levels of fibronectin and suppress the development of renal fibrosis.

There is a significant levels of fibronectin in normal human renal tissues. Previously, we found that levels of MAP1S are dramatically reduced in renal tissues from patients suffering with ccRCC and established that the impairment of MAP1S-mediated autophagy turnover of lipid droplets leads to the development of ccRCC [27]. Here, we observed that the levels of MAP1S in renal tissues from patients with renal atrophy and renal failure are dramatically reduced. Interestingly, the levels of fibronectin are dramatically elevated in renal tissues exhibiting obvious renal fibrosis. Combining the data from culture cells and mouse models, we conclude that MAP1S-mediated autophagy facilitates the degradation of fibronectin and MAP1S deficiency causes renal fibrosis in patients. We recently reported that the stability of MAP1S is directly regulated by HDAC4, a lysine deacetylase [33]. HDAC4 inhibition has demonstrated significant effects to increase the stability of MAP1S, MAP1S-mediated autophagy flux and degradation of mutant Huntingtin aggregates that are directly impact Huntington's disease [33]. Similar approaches to restore MAP1S-mediated autophagy flux in patients to reverse renal fibrosis should be feasible and promising.

## MATERIALS AND METHODS

### Antibodies, plasmids and other reagents

Monoclonal antibody against MAP1S (Cat# AG10006) was a gift from Precision Antibody™, A&G Pharmaceutical, Inc.. Primary antibodies against *β-actin* (SC-47778) and *GFP* (SC-8334) were purchased from Santa Cruz Biotechnology, Inc.. Antibodies against fibronectin (ab2413), TGF-β (ab66043) and α-SMA (ab-5694) were from abcam. Horseradish peroxidase-conjugated secondary antibodies against mouse (#172-1011) and rabbit (#172-1019) were from Bio-Rad. Rhodamine Red-X goat anti-mouse IgG (R6393) and FITC rabbit anti-mouse IgG (A21202) were from Invitrogen. RFP-LC3 was a gift from Dr. Mizushima [34]. Antibody against caspase 1 (PRS3459), bafilomycin A1 and Sirius Red (Direct Red 80, 365548) were from Sigma.

### Enrollment of patients and collection of human renal tissue samples from patients with renal fibrosis and renal failure

This study was approved by the institutional review boards of all participating sites, and these sites provided the necessary institutional data and shared agreements before study initiation. Six patients enrolled in Department of Urology, The Fifth Affiliated Hospital of Guangzhou Medical University from February 2010 to June 2015, were diagnosed as renal atrophy by ultrasound or CT examination and their renal function was further confirmed by radionuclide renal scan. Patients with glomerular filtration rate less than 10% were considered renal failure. Renal tissues were resected from the patients diagnosed as renal atrophy and renal failure. Six control samples were the normal tissues distant from tumor foci from six randomly selected patients who were enrolled in the same department during the same period and diagnosed as clear cell renal cell carcinomas. To pathologically confirm the diagnosis of renal fibrosis, the collected renal tissues were fixed in 10% formalin, embedded in paraffin, sectioned consecutively at 5 μm, and stained by hematoxylin and eosin by two independent clinical pathologists in a double-blinded manner. Additionally, the tissue sections were immuno-fluorescently stained to detect the levels of fibronectin and MAP1S following similar protocols as we previously described [5, 27].

### Culture of renal cells for immunoblot analyses

HK-2 (ATCC^®^ CRL-2190^™^) is a human papillomavirus 16 (HPV-16) transformed proximal tubular cell line derived from a normal kidney. Cells were cultured using standard techniques and harvested for immunoblot analyses as previously described [27, 35].

### Collection of murine renal tissues for analyses of renal fibrosis

Animal protocols were approved by the Institutional Animal Care and Use Committee, Institute of Biosciences and Technology, Texas A&M Health Science Center. All animals received humane care according to the criteria outlined in the “Guide for the Care and Use of Laboratory Animals” prepared by the National Academy of Sciences and published by the National Institutes of Health (NIH publication 86-23 revised 1985). Wild-type (MAP1S^+/+^) and MAP1S knockout (MAP1S^−/−^) mice expressing a single copy of GFP-LC3 or not were generated and amplified in a C57BL/6 background as described in detail in our previous publication [5, 22]. Male mouse littermates at different ages were sacrificed to collect renal tissues for immunofluorescent analysis with a confocal microscopy, immunoblot analyses, or Sirius Red staining as we previously described [5, 27, 35].
